# Modified use of a 4-branched frozen elephant trunk prosthesis for treatment of a right-sided aortic arch aneurysm with an aberrant left subclavian artery

**DOI:** 10.1093/icvts/ivab349

**Published:** 2021-12-24

**Authors:** Jens Brickwedel, Hermann Reichenspurner, Tilo Kölbel, Christian Detter

**Affiliations:** 1 Department of Cardiovascular Surgery, University Heart & Vascular Centre Hamburg, Hamburg, Germany; 2 Department of Vascular Medicine, University Heart & Vascular Centre Hamburg, Hamburg, Germany

**Keywords:** Right aortic arch, Frozen elephant trunk, Kommerell’s diverticulum, Aberrant subclavian artery

## Abstract

Right aortic arch aneurysm originating from a Kommerell’s diverticulum associated with an aberrant left subclavian artery is a rare congenital entity. We report a case of an asymptomatic 60-year-old female with right aortic arch aneurysm with an aberrant left subclavian artery, treated with a modified frozen elephant trunk technique using a 4-branched prosthesis, with the perfusion branch as an extra-anatomical bypass to the aberrant left subclavian artery. This case demonstrates short-term safety and efficacy of this technique.

## INTRODUCTION

Right aortic arch (RAA) aneurysm arising from a Kommerell’s diverticulum (KD) associated with an aberrant left subclavian artery (ALSA, lusorian artery) is a congenital rarity affecting 0.10% of the population [1]. Established guidelines for the appropriate surgical technique are non-existent. We report the modified frozen elephant trunk (FET) technique for RAA aneurysm with an ALSA [2].

## CASE PRESENTATION

An asymptomatic, 60-year-old female presented with a distal RAA aneurysm, an incidental detection during diagnostic work-up for Covid-19 negative lobular pneumonia.

**Figure 1:**
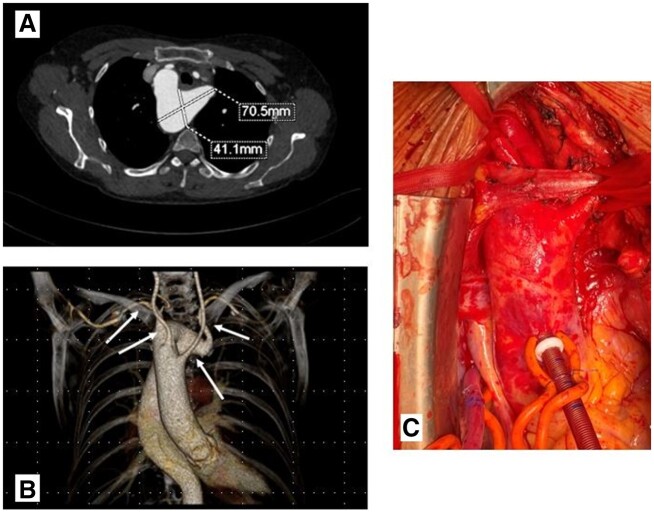
(**A**) Contrast computed tomography of Kommerell’s diverticulum, (**B**) computed tomography three-dimensional reconstruction: 4 vessels arising individually and (**C**) intraoperative anatomy: looped right subclavian artery, right common carotid artery, left common carotid artery and brachiocephalic vein.

Computerized tomography angiography revealed: (i) an ALSA originating from a KD with distal RAA aneurysm of 70 mm; (ii) Four arteries arising individually from the RAA, proximal to distal: left common carotid, right common carotid, right subclavian (RSA) and the ALSA from the KD crossing left behind trachea and oesophagus; non-dilated ascending and lower descending aorta (Fig. 1).

A unanimous decision was taken by the multidisciplinary aortic team to perform a modified FET procedure. Fenestrated endovascular treatment was not feasible due to a missing proximal landing zone. Preoperative investigations revealed chronic obstructive pulmonary disease GOLD stage II as relevant comorbidity.

**Figure 2:**
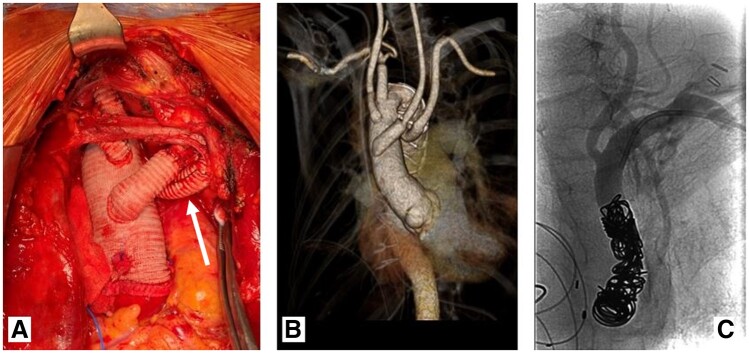
(**A**) Prosthetic branches anastomosed to right subclavian artery, right common carotid artery and left common carotid artery, perfusion branch to aberrant left subclavian artery bypass (arrow), (**B**) postoperative computed tomography and (**C**) aberrant left subclavian artery angiography after coiling.

Surgery commenced with ALSA exposure via a left-sided supraclavicular approach. An 8-mm Dacron graft (Gelweave™ prosthesis, Terumo Aortic, Glasgow, UK) was anastomosed to the ALSA and cannulated using a 22-Fr arterial cannula for full body perfusion. Median sternotomy was performed and cardiopulmonary bypass (CPB), lasting 353 min, established after right atrium and additional ascending aortic cannulation (Fig. 1C). The patient was cooled to 25°C. Circulatory arrest for 126 min and retrograde cold blood cardioplegia was commenced. Catheters were inserted into the left common carotid artery and right common carotid artery for bilateral antegrade cerebral perfusion for 209 min. The aortic arch was transected between the origin of the ALSA and right subclavian artery. The ALSA origin was anatomically inaccessible for direct surgical closure, thus a 30/36 mm FET prosthesis with a 100-mm stent-graft (4-branched Thoraflex Hybrid graft, Vascutek, Inchinnan, Scotland, UK) was inserted and deployed, covering the ALSA origin and excluding the KD. Perfusion branch was cannulated and lower body perfusion commenced after distal graft anastomosis in zone 2. The third, second and first branches of the Hybrid prosthesis were anastomosed to the right subclavian artery, right common carotid artery and left common carotid artery, respectively. Subsequently, selective cerebral perfusion was completed. During rewarming, the proximal prosthetic end was anastomosed to the ascending aorta, aortic clamp removed and myocardial reperfusion started after de-airing. Weaning from CPB was uneventful. A retro-clavicular tunnel between the left supraclavicular incision and upper thoracic aperture was created by blunt dissection [2]. The ALSA graft previously used for arterial inflow was pulled downwards below the left sternoclavicular joint into the anterior mediastinum. The FET perfusion branch was not ligated, but anastomosed end-to-end to the ALSA graft instead (Fig. 2). Heparin was antagonized with protamine. The postoperative in-hospital stay was prolonged due to chronic obstructive pulmonary disease, but otherwise uneventful, except for a right recurrent nerve palsy. The patient was discharged 14 days postoperatively. Prior to discharge, postoperative computed tomography (Fig. 2B) revealed good position and distal seal of the FET with unimpeded perfusion of the supra-aortic vessels and partial proximal ALSA thrombosis. Proximal ALSA was occluded 3 months postoperatively under local anaesthesia by coil embolization (Fig. 2C).

## DISCUSSION

The RAA with KD aneurysm greater than 66 mm reported in our case is rare, with no established treatment recommendations. Current options include surgical versus endovascular treatment. Barr *et al.* proposed a patient-tailored surgical treatment algorithm [1, 3, 4].

To our knowledge, this is the first reported case where the perfusion branch of the Thoraflex prosthesis was used in a modified FET technique, not only for CPB perfusion but as an extra-anatomical bypass to the ALSA as well, after weaning from CPB.

Based on our experience with this rare case, the modified FET technique demonstrates short-term safety and efficacy and is a valuable treatment option.

**Conflict of interest**: none declared. 

## Reviewer information

Interactive CardioVascular and Thoracic Surgery thanks Anthony L. Estrera and the other anonymous reviewers for their contribution to the peer review process of this article.
